# Reflex Paralysis: Its Pathological Anatomy and Relations to the Sympathetic Nervous System

**Published:** 1866-08

**Authors:** 


Reflex Paralysis: Its Pathological Anatomy and Relations to the Sympathetic Nervous System. By M. Gonzales Echeverria, M.D., Physician to the Charity Hospital, New York; formerly Assistant-Physician to the National Hospital for the Paralyzed and Epileptic, of London, etc. Bailiere & Brothers, New York. 1866.

This is an excellent monograph of 80 pages. The greater part of it originally appeared in a series of articles in the *New York Medical Journal.*

